# Evaluation of *Abelmoschus moschatus *extracts for antioxidant, free radical scavenging, antimicrobial and antiproliferative activities using *in vitro *assays

**DOI:** 10.1186/1472-6882-11-64

**Published:** 2011-08-17

**Authors:** Mir Z Gul, Lepakshi M Bhakshu, Farhan Ahmad, Anand K Kondapi, Insaf A Qureshi, Irfan A Ghazi

**Affiliations:** 1 Department of Plant Sciences, School of Life Sciences, University of Hyderabad, Gachibowli, Hyderabad 500 046, India; 2 Department of Biotechnology, School of Life Sciences, University of Hyderabad, Gachibowli, Hyderabad 500 046, India

## Abstract

**Background:**

*Abelmoschus moschatus *Medik. leaves and seeds are considered as valuable traditional medicine. The aromatic seeds of this plant are aphrodisiac, ophthalmic, cardio tonic, antispasmodic and used in the treatment of intestinal complaints and check queasiness. To give a scientific basis for traditional usage of this medicinal plant, the seed and leaf extracts were evaluated for their antioxidant, free radical scavenging, antimicrobial and antiproliferative activities.

**Methods:**

In this study, antioxidant, antimicrobial and antiproliferative activities of *A. moschatus *extracts were evaluated in a series of *in vitro *assay involving free radicals, reactive oxygen species and their IC_50 _values were also determined. The antioxidant activities of the seed and leaf extracts of *A. moschatus *were determined by total antioxidant, DPPH, and ferrous reducing antioxidant property (FRAP) methods. In addition, the antiproliferative activity was also evaluated using colorectal adenocarcinoma and retinoblastoma human cancer cell lines. Moreover, six bacterial reference strains, two gram-positive (*Bacillus subtilis *and *Staphylococcus aureus*), four gram-negative (*Escherichia coli, Pseudomonas aeruginosa, Proteus vulgaris *and *Salmonella enterica paratyphi*) and one fungal strain (*Candida albicans*) were used to evaluate its antimicrobial activity.

**Results:**

The results from this study showed that the antioxidant activities of *A. moschatus *as determined by the total phenol, flavonoids, total antioxidant and FRAP methods were higher in leaf than that of the seed extracts. On the other hand, the aqueous overnight seed extract (AMS-I) has shown significant radical scavenging activity as in 1, 1- Diphenyl-2-picrylhydrazyl (DPPH), hydrogen peroxide, hydroxyl radical, superoxide and lipid peroxidation as compared to other seed and leaf extracts. The AMS-I and AML-IV have shown activity against six and seven microorganisms respectively. Simulteneously, AMS-IV and AML-IV have demonstrated potential antiproliferative activity against two human cell lines - Colorectal adenocarcinoma (COLO-205) and retinoblastoma (Y79).

**Conclusion:**

The seed and leaf extracts of *A. moschatus *possess significant antioxidant activity and could serve as free radical inhibitors or scavenger, or substitute, probably as primary antioxidants. The plant possesses moderate antibacterial activity against bacterial strains used in this study. Hydroalcoholic seed and leaf extracts also exhibited antiproliferative activity against two human cancer cell lines. *A. moschatus *may therefore, be a good candidate for functional foods as well as pharmaceutics.

## Background

The free radicals (FR) and reactive oxygen species (ROS) are produced through frequent physiological and biochemical processes in the human body as byproduct [1,2]. ROS includes a number of chemically reactive molecules derived from oxygen, such as hydrogen peroxide (H_2_O_2_), superoxide (O_2_**^-^**) and hydroxyl radical (OH**^-^**) etc. Over production of such free radicals might leads to oxidative damage of biomolecules in the body (e.g. lipids, proteins, DNA) that can initiate number of diseases like atherosclerosis, diabetes mellitus, cancer, heart and neurodegenerative diseases etc. [3,4]. The harmful effect of the free radicals can however, be blocked by antioxidant substances. Plants produce wide array of secondary metabolites such as phenolic compounds (phenolic acids, flavonoids, quinines and coumarins), nitrogen compounds (alkaloids and amines), vitamins, terpenoids and other secondary metabolites that have been proved for antioxidant activities [5,6]. Current research has confirmed that antioxidants are the most effective tools to eliminate free radicals which cause oxidative stress and are possible protective agents that protect the cells from ROS and retard the progress of many diseases as well as lipid peroxidation [7-9]. Moreover, in recent past, the polyphenols have found to be beneficial as strong antioxidants [10]. In this context, evaluation of the polyphenols and antioxidant activity in herbs has become important tool to understand the healing property of medicinal plants.

Natural products from the medicinal plants provide unlimited opportunities for new drugs because of the unparalleled accessibility of diverse chemical compounds [11]. Cancer is a leading cause of death worldwide and it accounted for 7.9 million deaths (around 13% of all deaths) in 2007. It is also reported that more than 70% of all deaths of cancer occurred in middle and low income countries. Anticancer drugs from natural sources such as plants, marine organisms and microorganisms account approximately 60% of all anticancer drugs [12]. For thousands of years, human beings have used natural substances especially plants to relieve pain, heal wound and maintain health. Most of the bioactive components in medicinal plants probably evolved as chemical defence agents against infections or predators. Many plants were proved to be very important source of anticancer agents due to reducing risk factors of the cancer by consuming vegetables and fruits which are rich in naturally occurring phytochemicals including phenols and flavonoids [13]. Several investigations were carried out to evaluate anticancer properties of herbs and are being used as potent anticancer drugs [14]. In addition to antioxidant activity, the valuable health benefits of different medicinal plants were claimed as antibiotic agents against pathogenic microorganisms. There is also an urgent necessity to develop alternative antimicrobial drugs for the treatment of infectious diseases from medicinal plants [15]. The antimicrobial compounds generated by these medicinal plants are active against plant and human pathogenic microorganisms [16]. They are efficient in the treatment of infectious diseases and at the same time mitigate many of the side effects namely, hypersensitivity, immune-suppression and allergic reactions that are often associated with synthetic antimicrobials [17]. Moreover, there is an alarming prevalence of antibiotic resistance in bacteria of medical importance [18].

Keeping in view of the demand for developing natural antioxidants, effective antiproliferative and antimicrobial drugs, the present study was aimed to investigate the antioxidant, anti free radical, antimicrobial and antiproliferative activities of different extracts of *A. moschatus*, which belongs to family, malvaceae and popularly known as Mushkdana/Kasturi bhendi. The selection of this plant for evaluation was based on its traditional usage. A survey of the literature revealed that the seeds of this plant (powdered form in lukewarm milk) have been recommended for use in various traditional systems of medicine for the treatment of intestinal complaints, constipation, dyspepsia and gonorrhea. The seeds are used as stimulant, relaxant and also for casting out the poison of snakes. The seeds also serve as cardiac tonic, aphrodisiac, diuretic, antispasmodic. Moreover, the leaf decoction has been effective against intestinal complaints and checks vomiting. The tincture of leaf powder is applied for skin diseases [19]. Thus, our main objective of the present investigation was to evaluate the antioxidant, antiproliferative and antibacterial potential of this plant, in addition to quantifying the polyphenols of extracts, which might be responsible for biological activities.

## Methods

### Chemicals

All the chemicals were purchased from Hi-Media and Merck, India. Standard drugs were purchased from Sigma-Aldrich chemicals co. (St. Louis O., USA); and RPMI-1640, DMEM & serum from Gibco, (Invitrogen, USA). Analytical grade solvents were used in this study.

### Plant material

Seeds and healthy leaves of *A. moschatus *were collected from the Central Institute of Medicinal and Aromatic Plants (CIMAP), Regional Centre Hyderabad, India during the month of September-October, 2009. The seeds and leaves were cleaned, dried under shade, ground to a coarse powder and stored in an air-tight container at 25°C for further use.

### Microbial cultures

Bacterial reference strains *Bacillus subtilis *ATCC 5740, *Staphylococcus aureus *ATCC 25923, *Escherichia coli *ATCC 25922, *Pseudomonas aeruginosa *ATCC 27853, *Proteus vulgaris *ATCC 6380, *Salmonella enterica paratyphi (Salmonella paratyphi*) ATCC 9150 and *Candida albicans *ATCC 10231 were obtained from Central Research Institute of Unani Medicine, Hyderabad, India. The pure cultures were maintained on nutrient agar slants for the entire study. All the isolates were sub-cultured at regular time period and stored at 4°C as well as at -80°C by making their suspension in 10% glycerol.

### Preparation of plant extracts

The seed and leaf powder of *A. moschatus *were subjected to different modes of extraction using ethanol and water in order to find out the suitable extract with maximum biological activities. Aqueous extracts of *A. moschatus *seed (AMS-I) and leaf (AML-I) were prepared by soaking 1 g of dried powder in 4 mL of distilled water for 24 h at room temperature. Other aqueous extracts (AMS-II and AML-II) were prepared using dried powder of seed and leaf (1 g each) through slow evaporation at 30-40°C. Also decoctions were prepared from the seed and leaf powder (1 g each) by mixing with 20 mL distilled water for 3-4 h at 80-90°C and the extracts were designated as AMS-III and AML-III respectively.

Hydroalcoholic extracts of seed (AMS-IV) and leaf (AML-IV) were prepared by dissolving the dried powder of seed and leaf (1 g each) in 20 mL of 80% (v/v) ethanol for 3-4 h at 40-50°C and were evaporated to 4 mL. The suspensions prepared in all above cases were centrifuged at 10,000 rpm for 15 min. The supernatant were collected into separate tubes and concentrated to the dry mass using vacuum evaporator. The residues were stored in amber glass bottles at -20°C for further analysis. The dried extracts of 20 mg/mL stock solution were prepared and different concentrations were used in the experiments.

### Determination of total phenolic content

The amount of total soluble phenolic content in different seed and leaf extracts was determined according to Folin-Ciocalteu method [20] with slight modifications. Briefly, 10 μL of extract solution from the stock solution was mixed with 100 μL of Folin-Ciocalteu reagent. After 10 min of incubation, 300 μL of 20% Na_2_CO_3 _solution was added and the volume was adjusted to 1 mL using distilled water. The mixture was incubated in dark for 2 h and the absorbance was measured at 765 nm using a UV-Vis spectrophotometer against blank sample. The total phenolic content was measured as gallic acid equivalents (mg GAE)/gram of dry weight (dw) and the values were presented as means of triplicate analysis.

### Determination of total flavonoid content

Total flavonoid content was estimated by a colorimetric method [21] by taking 20 μL of each extract and mixed with 500 μL Milli-Q water and 30 μL of 5% NaNO_2 _solution. After 5 min of incubation at room temperature, 60 μL of 10% AlCl_3 _solution was added. Subsequently, 350 μL of 1 M NaOH and 40 μL of Milli-Q water were added to make the final volume 1 mL. Samples were further incubated for 15 min at room temperature and the absorbance of the samples was measured at 510 nm. The total flavonoids were determined as qurecetin equivalents (mg QE)/g of dw and the values were expressed as means of triplicate analysis.

### Evaluation of antioxidant capacity

#### Determination of total antioxidant activity (TAA)

The total antioxidant activity of both seed and leaf extracts of *A. moschatus *were evaluated by phosphomolybdenum method [22]. The assay is based on the reduction of Mo (VI) - Mo (V) by the antioxidant compounds and subsequent formation of a green phosphate/Mo (V) complex at acidic pH. Different extracts of 10 μL each from the stock solution were dissolved in 90 μL distilled water and 1 mL of reagent solution (0.6 M sulphuric acid, 28 mM sodium phosphate and 4 mM ammonium molybdate) in 1.5 mL tubes. The tubes were capped and incubated in a thermal block at 95°C for 90 min. After cooling to room temperature, the absorbance of the solution of each reaction was measured at 695 nm against blank samples. Ascorbic acid (AA) was used as standard and the total antioxidant capacity was expressed as milligrams of ascorbic acid equivalents (mg AAE/g) of dw.

#### Determination of reducing antioxidant power (FRAP)

The ferric ions (Fe^3+^) reducing antioxidant power (FRAP) method [23] was used to measure the reducing capacity of seed and leaf extracts with a slight modification which involves the presence of extracts to reduce the ferricyanide complex to the ferrous form. The FRAP method is based on a redox reaction in which an easily reduced oxidant (Fe^3+^) is used in stoichiometric excess and antioxidants acts as reductants. Various concentrations of extracts (seed and leaf) of *A. moschatus *from the stock solutions and the standard (ascorbic acid) were mixed with 2.5 mL of phosphate buffer (0.2 M, pH 6.6) and 2.5 mL of potassium ferricyanide (1% w/v). The mixture was incubated at 50°C for 20 min. Then 2.5 mL of trichloroacetic acid (10% w/v) was added to the reaction mixture, which was then centrifuged at 1000 g for 10 min. The upper layer of the solution (2.5 mL) was mixed with deionised water (2.5 mL) and ferric chloride (0.5 mL, 0.1% w/v). The absorbance was measured at 700 nm at the reaction time of 30 min. The reducing power of the extracts was represented as mg AAE/g of dw.

#### DPPH radical scavenging activity

The antioxidant activity of the plant extracts was assessed on the basis of the radical scavenging effect using stable 1,1-diphenyl-2-picrylhydrazyl (DPPH) [24]. DPPH solution (0.004% w/v) was prepared in 95% methanol and serial dilutions were carried out with the stock solutions (20 mg/mL) of the extracts. Various concentrations of extracts were mixed with DPPH solution (900 μL), incubated in dark for 30 min and then absorbance was measured at 517 nm. Methanol (95%), DPPH solution and ascorbic acid (AA) were used as blank, control and reference standard respectively.

#### Hydrogen peroxide scavenging activity

Hydrogen peroxide scavenging activity was determined according to a ferrous ion oxidation - xylenol orange (FOX) assay [25] with minor changes. FOX reagent was prepared by adding nine volumes of reagent 1 to one volume of reagent 2, where reagent 1 was 4.4 mM butylated hydroxytoluene (BHT) in methanol and reagent 2 was 1 mM xylenol orange and 2.56 mM ammonium ferrous sulfate in 250 μM H_2_SO_4_. Plant extracts of different concentrations were incubated with 10 μL of 40 mM H_2_O_2 _for 10 min at room temperature in dark and 0.2 mL of FOX reagent was added and the volume was made upto 1 mL with distilled water. The reaction mixture was then vortexed and incubated at room temperature for 30 min. Development of violet colour indicates control reaction and discoloration was considered as scavenging activity after the addition of plant extracts or standard (ascorbic acid). The FOX reagent without extracts/H_2_O_2 _served as blank and with H_2_O_2 _served as control. The absorbance of the ferric-xylenol orange complex was measured at 560 nm.

#### Superoxide radical scavenging activity

The superoxide radical scavenging activity of seed and leaf extracts of *A. moschatus *was performed according to the method given by Kakkar *et al. *[26] with minor modifications. Briefly, solutions containing 156 μM nitroblue tetrazolium (NBT) dissolved in 50 mM phosphate buffer (pH 7.4), 468 μM nicotinamide adenine dinucleotide (NADH) and various concentrations of extracts were mixed. The reaction was started by addition of 100 μL of 60 μM phenazine methosulfate (PMS) solution and the final volume of the reaction was 3 mL. The reaction mixture was incubated at 25°C for 5 min and absorbance at 560 nm was observed against control samples (with NADH).

#### Hydroxyl radical scavenging activity

Hydroxyl radical scavenging activity was measured as per the protocol of Kunchandy and Rao [27] with minor changes by studying the competition between deoxyribose and test extracts for hydroxyl radicals generated by Fenton's reaction. Briefly, solution of Fenton's reagent [Fe (III) chloride, ascorbic acid and H_2_O_2_] was prepared in distilled water just prior to use. To 0.1 mL Fenton's reagent, thiobarbituric acid (1% w/v) in 25 mM NaOH (1 mL) and tricholoroacetic acid (1 mL, 2.8% w/v) were added and volume was made to 3 mL with distilled water. The mixture was heated for 90 min on water bath at 80°C and the amount of pink chromogen produced was considered as control. Finally it was measured spectrophotometrically at 532 nm. The protection of oxidation of D-ribose has been conducted by pre-incubation with the *A. moschatus *extracts in different concentrations and decrease in the formation of pink colour was considered as antioxidant property which was compared to the standard ascorbic acid.

#### Inhibition of Fenton's reagent-induced strand breaks in plasmid DNA

The ability of different extracts to protect super coiled pBR322 DNA from devastating effects of hydroxyl radicals generated by Fenton's reagent was assessed by DNA nicking assay [28] with minor modifications. The reaction mixture contained 2.5 μL of plasmid DNA (0.25 μg) and 10 μL Fenton's reagent (30 mM H_2_O_2_, 500 μM ascorbic acid and 800 μM FeCl_3_) followed by the addition of 5 μL of extracts and the final volume of the mixture was brought upto 20 μL with distilled water. The reaction mixture was then incubated for 45 min at 37°C and analyzed on 0.9% agarose gel electrophoresis by staining with ethidium bromide.

#### Determination of inhibition of Lipid peroxidation

Lipid peroxidation inhibitory activity of *A. moschatus *extracts and the standard (ascorbic acid) were carried out according to the standard protocol [29]. The rat liver homogenate was used for induction of lipid peroxidation, mediated by FeCl_3 _as pro-oxidant. Healthy albino rats of the wister strain (250 g) were sacrificed and perfused the liver with 0.15 M KCl and homogenate was centrifuged at 800 g for 15 min at 4°C and the supernatant was used for the thiobarbutaric acid assay. The extracts of *A. moschatus *at different concentrations were mixed with the liver microsome preparation and the mixtures were incubated in the presence and absence of Fenton's reagent (50 μL of 10 mM FeCl_3_; 10 μL of 2.5 mM H_2_O_2_) in phosphate buffer (0.2 M, pH 7.4) and the final volume was made to 1 mL. The reaction mixtures were incubated at 37°C for 30 min. After incubation, 2 mL of ice-cold HCl (0.25 N) containing 15% trichloroacetic acid, 0.5% thiobarbutaric acid, and 0.5% butylated hydroxytoluene (BHT) was added to the reaction mixture, followed by heating at 100°C for 60 min. The reaction mixture was put in an ice bath for 10 min. The mixture was centrifuged at 1000 g for 10 min and the extent of lipid peroxidation was subsequently monitored by the formation of thiobarbutaric acid reactive substances (TBARS) as pink chromogen in the presence or absence of extracts and standard (ascorbic acid). The absorbance of the supernatant was measured spectrophotometrically at 532 nm. The decline in the formation of pink chromogen in the pretreated reactions was considered as inhibition of lipid peroxidation.

#### Antiproliferative activity

For the assessment of the antiproliferative activity of plant extracts, two human tumor cells, colorectal adenocarcinoma (COLO-205) and retinoblastoma (Y79) cells were used. The cell lines were purchased from National Centre for Cell sciences (NCCS), Pune, India. The antiproliferative activity of the selected cell lines was performed and the reduction of 3- (4, 5- dimethylthiozol-2-yl) - 2, 5- diphenyltetrazolium bromide was chosen as an optimal end point of cell viability measurement. COLO-205 and Y79 cells (0.2 × 10^6 ^cells per well) were grown in DMEM and RPMI 1640 respectively, alongwith 10% Fetal bovine serum **(**FBS) in 96-well plates. Increasing concentrations (25, 50, 100, 200 μg) of seed and leaf extracts of *A. moschatus *dissolved in 10% Dimethyl sulfoxide (DMSO) were added to the cells (final concentration of DMSO was 2%) and incubated at 37°C under 5% CO_2 _in a humidified incubator for 14 h. The cell suspension was centrifuged at 1000 g for 10 min and the medium was aspirated. Subsequently, the fresh growth medium containing 20 μL of MTT solution of 5 mg/mL was added to each well [30]. After incubation for 4 h in a humidified atmosphere, the medium was removed by centrifugation at 1000 g for 10 min and 200 μL of DMSO was added to the wells to dissolve the MTT-formazan crystals. The plates were shaken and absorbance was determined by ELISA reader (TECAN) at 570 nm. The conventional anticancer drug, ifosfamide was used as a positive control and 2% DMSO as solvent control. Controls and samples were assayed in triplicates for each concentration and replicated three times for each cell line. The cytotoxicity was obtained by comparing the absorbance between samples and controls.

#### Antimicrobial activity

The seed and leaf extracts of *A. moschatus *were tested against the reference strains for antimicrobial activity using micro dilution method in 96 well microtiter plates [31] with minor modifications and recommended by the National Committee for Clinical Laboratory Standard [32]. The antimicrobial activity of the extracts was evaluated against two gram positive (*B. subtilis *ATCC 5740, *S. aureus *ATCC 25923), four gram negative (*E. coli *ATCC 25922, *P. aeruginosa *ATCC 27853, *P. vulgaris *ATCC 6380, *S. enterica paratyphi *ATCC 9150) bacterial strains and one fungal strain (*C. albicans *ATCC 1023). Briefly, antimicrobial activity was carried out in 96 well microtiter plate containing different concentrations of extracts. The culture suspension (100 μL) was added to each well having 10^5 ^CFU/mL and final volume was made to 200 μL by adding LB broth. Plates were incubated at 37 ± 1°C for 18 h and then 10 μL of MTT (5 mg/mL) was added to each well. The plates were examined with ELISA reader (TECAN) at 530 nm and the lowest concentration of each extract which showed complete inhibition was taken as its minimum inhibitory concentration (MIC). In control experiments, sterile distilled water and ethanol were added in place of plant extracts; whereas, antibiotics such as ampicillin, kanamycin and nystatin (1 mg/mL) were used as positive controls. For blank reaction, the sterile broth was used in place of suspension cultures (without inoculums).

### Calculations and Statistical analysis

The percentage inhibition of free radicals, lipid peroxidation and cytotoxic activities of the extracts were calculated using the formula:

$$\%\;\text{Inhibition}=\left[\left(\text{A}\;\text{control}-\text{A}\;\text{sample}\right)∕\text{A}\;\text{control}\right]\times 100$$

All analyses were performed in triplicates. The experimental results were expressed as mean ± standard deviation of mean (SEM) of three replicates. The concentration of the extract that was required to scavenge 50% of radicals (IC_50_) was calculated for different seed and leaf extracts of *A. moschatus*. The graphical representation of the results was done using Sigma -11 software.

## Results

### Total phenolic and flavonoid content

The results of total phenolic content of different seed and leaf extracts of *A. moschatus *were significant and shown in Table 1. The total phenol content in the seed extracts (AMS-I, II, III and IV) expressed as gallic acid equivalent (GAE) were in the range of 1.56 to 3.74 mg GAE/g dw. AMS-I had the highest content as 3.74 mg GAE/g dw, whereas AMS-IV contained a much smaller amount as 1.56 mg GAE/g dw. In leaf extracts, the total phenol content was significantly higher compared to seed extracts and varied from 9.49 to 13.84 mg GAE/g dw, AML-IV extract showed higher level of total polyphenol content (13.84 mg GAE/g dw), whereas the lowest content of total polyphenol was found in AML-I (9.49 mg of GAE/g dw).

**Table 1:** Total polyphenol, flavonoid, antioxidants and ferric reducing antioxidant power of *A. moschatus *extracts

Extract	**Polyphenols**^**a**^	**Flavonoids**^**b**^	**Antioxidants**^**c**^	Ferric reducing ** antioxidant power**^**d**^
AMS-I	3.74 ± 0.13	0.10 ± 0.02	10.78 ± 0.16	0.54 ± 0.05
AMS-II	2.35 ± 0.08	0.13 ± 0.08	8.89 ± 0.04	0.46 ± 0.03
AMS-III	1.73 ± 0.02	0.22 ± 0.03	9.12 ± 0.06	0.38 ± 0.04
AMS-IV	1.56 ± 0.02	0.26 ± 0.02	8.08 ± 0.08	0.42 ± 0.04
AML-I	9.49 ± 0.17	5.60 ± 0.02	13.30 ± 0.33	3.02 ± 0.05
AML-II	11.86 ± 0.11	2.00 ± 0.08	15.30 ± 0.15	4.51 ± 0.04
AML-III	13.38 ± 0.26	3.12 ± 0.03	19.85 ± 0.07	6.07 ± 0.02
AML-IV	13.84 ± 0.10	6.00 ± 0.02	21.52 ± 0.07	6.28 ± 0.01

a: gallic acid; b: quercetin; c & d: ascrobic acid equivalents mg/g dw plant material respectively; Results represented in means ± standard deviation (n = 3).

Total flavonoid content of the seed and leaf extracts was recorded in least quantities in quercetin equivalents (QE) and in comparison to the total phenolics (Table 1). All the four extracts of seeds (AMS-I, AMS-II, AMS-III and AMS-IV) contained total flavonoids in minimum amount, highest being in AMS-IV (0.26 mg QE/g dw). The leaf extracts also contained some flavonoid content with the highest value observed in AML-IV (6.0 mg QE/g dw). The overall levels of total polyphenol and flavonoid content in the plant extracts were found significantly lower when compared to the standard compounds used in this study.

### Total antioxidant activity (TAA) and ferric reducing antioxidant power (FRAP)

The extracts of seed and leaf exhibited significant antioxidant activity, thus establishing the extracts as an antioxidant. The results of the antioxidant measurements are summarized in Table 1. The antioxidant activity was in the range of 8.08 to 10.78 mg AAE/g dw in the seed extracts. The highest value of 10.78 mg AAE/g dw was observed in AMS-I whereas the lowest value (8.08 mg AAE/g dw) was found in AMS-IV. The leaf extracts of *A. moschatus *showed reasonably higher antioxidant activity in comparison to the seed extracts. The activity was in the range of 13.30-21.52 mg AAE/g dw whereas AML-IV exhibited highest activity with value of 21.52 mg AAE/g dw and AMS-I with least activity 13.30 mg AAE/g dw.

The extracts of *A. moschatus *expressed electron donating activity, but their power was inferior to ascorbic acid, which is known to be a strong reducing agent (Table 1). Leaf extracts exhibited considerably higher reducing power for Fe^3+ ^than the seed extracts. The reducing ability of the leaf extracts was in range of 3.02-6.28 mg AAE/g dw. The highest value was observed in AML-IV (6.28 mg AAE/g dw), whereas the lowest value was recorded in AML-I (3.02 mg AAE/g dw). The FRAP values for the seed extracts were in the range of 0.38-0.54 mg AAE/g dw. AMS-I showed highest value of 0.54 mg AAE/g dw whereas AMS-III depicted least value (0.38 mg AAE/g dw).

### DPPH radical scavenging activity

**Figure 1:**
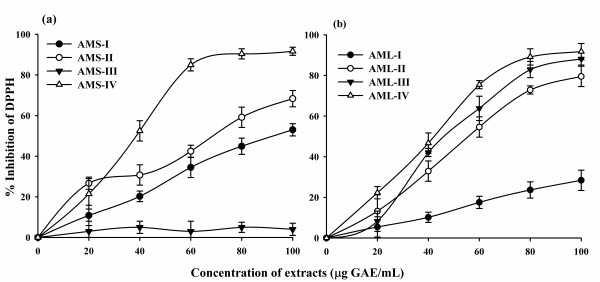
**DPPH scavenging activity of the *A. moschatus *seed (a) and leaf (b) extracts**.

In this study, all the extracts showed tendency to quench the DPPH free radicals, as indicated by the concentration dependent increase in percentage inhibition. The results revealed that the leaf extracts had the higher DPPH radical scavenging ability than those of the seed extracts. The IC_50 _values (concentration of the extract that was able to scavenge half of the DPPH radical) are presented in Table 2. Among the seed extracts, AMS-IV exhibited stronger radical scavenging ability and its percentage inhibition reached to 91.6% with the lowest IC_50 _value of 38.1 μg GAE/mL, which indicates its good antioxidant potential. The other seed extracts showed moderate DPPH radical scavenging effects (Figure 1a; Table 2). On the other hand, leaf extracts showed significantly stronger activities and quenched DPPH radicals to different degrees at higher concentrations. The scavenging activity reached to 91.7% with IC_50 _value of 42.8 μg GAE/mL in AML-IV, followed by AML-III. The lowest percentage of inhibition was observed in AML-I (28.4% with IC_50 _value of 176.1 μg GAE/mL) (Figure 1b; Table 2).

**Table 2:** IC_50 _values of *A. moschatus *extracts on tested radicals

Name of the Assay	Seed*				Leaf*				Standard†
		
	AMS -I	AMS -II	AMS -III	AMS -IV	AML -I	AML-II	AML-III	AML-IV	
DPPH	93.6 ± 3.0	70.7 ± 6.0	56.3 ± 15.0	38.1 ± 8.0	176.1 ± 14.0	58.5 ± 1.2	47.5 ± 1.0	42.8 ± 1.0	3.5 ± 0.2
Hydrogen peroxide	22.6 ± 5.0	26.3 ± 4.0	24.6 ± 10.0	138. ± 12.0	NA	NA	NA	NA	44.8 ± 0.4
Super oxide radical	22.3 ± 2.0	26.3 ± 3.0	28.4 ± 14.0	NA	30.6 ± 3.0	NA	NA	NA	25.5 ± 0.6
Hydroxyl radical	16.3 ± 2.0	18.5 ± 4.0	20.1 ± 12.0	22.8 ± 7.0	10.7 ± 3.0	18.7 ± 3.0	22.7 ± 4.0	22.4 ± 2.0	55.3 ± 0.8
Lipid peroxidation	76.2 ± 2.0	136.3 ± 8.0	146.3 ± 4.0	148.3 ± 6.0	60.5 ± 4.0	65.4 ± 3.0	85.4 ± 4.0	88.9 ± 4.0	45.2 ± 0.3

(*Values expressed in μg of GAEs/mL; **†: **Ascorbic acid in μg/mL; Results represented in means ± standard deviation (n = 3); NA: No activity.

### Hydrogen peroxide scavenging activity

**Figure 2:**
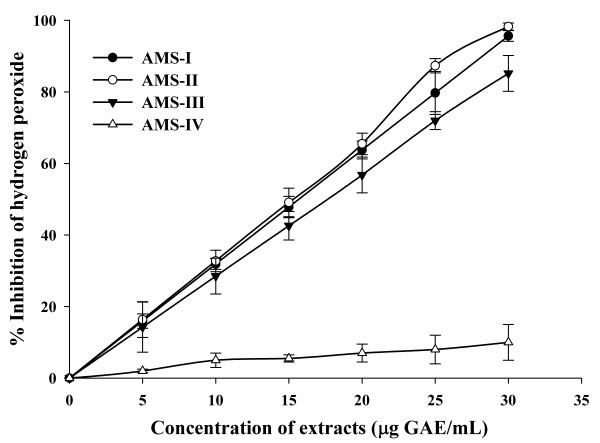
**Hydrogen peroxide scavenging activities of seed extracts of *A. moschatus***.

Among the seed and leaf extracts of *A. moschatus*, only three seed extracts (AMS-I, AMS-II and AMS-III) were capable of scavenging H_2_O_2 _in a concentration dependent manner and IC_50 _values for scavenging of H_2_O_2 _were 22.6, 26.3 and 24.6 μg GAE/mL respectively (Table 2; Figure 2). The IC_50 _for ascorbic acid was 44.8 μg GAE/mL. Since any of the leaf extracts did not show inhibition of the peroxide radical generation, therefore, no figure or IC_50 _values were provided.

### Superoxide radical scavenging activity

**Figure 3:**
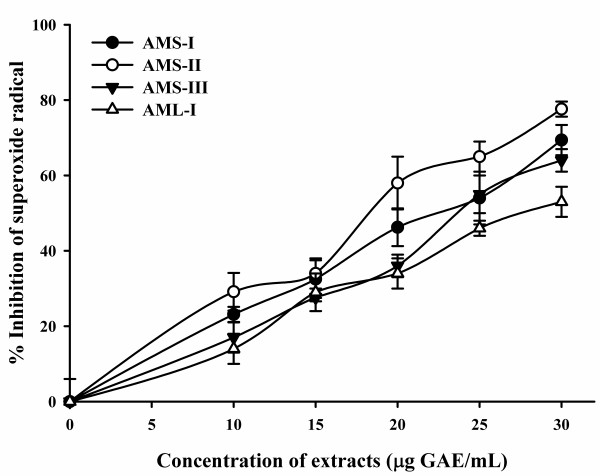
**Superoxide scavenging activities of seed extracts of *A. moschatus***.

The superoxide radical scavenging activity of *A. moschatus *extracts assayed by the PMS-NBT-NADH system was shown in Figure 3; Table 1. Three extracts of seed (AMS-I, AMS-II and AMS-III) and one leaf extract (AML-I) were found to be an efficient scavenger of superoxide radical generation. The maximum inhibition of 87.4% with IC_50 _value of 22.3 μg GAE/mL was observed in AMS-I, whereas AMS-II showed inhibition value of 69.9% with the IC_50 _value of 26.3 μg GAE/mL. The leaf extracts, AML-I inhibited superoxide radical upto 66.6% with IC_50 _value of 30.6 μg GAE/mL (Table 2; Figure 3). This result clearly indicated that the tested extracts had a noticeable effect on scavenging superoxide radical.

### Hydroxyl radical scavenging activity

**Figure 4:**
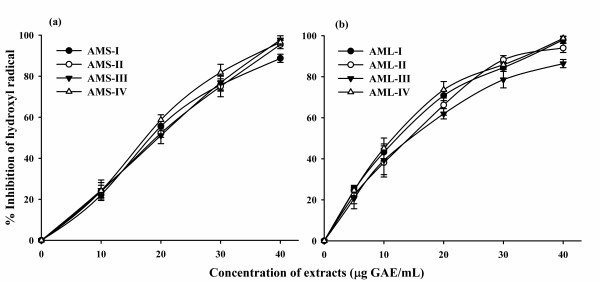
**Hydroxyl radical scavenging activities of the extracts of seed (a) and leaf (b) of *A. moschatus***.

This assay showed the abilities of the extracts and standard (ascorbic acid) to inhibit hydroxyl radical-mediated deoxy-ribose degradation. The *A. moschatus *seed and leaf extracts showed significant inhibition of hydroxyl radicals generated by Fenton's reagent in a concentration dependent manner. The OH^- ^radical scavenging data (Table 2) indicated that extracts of *A. moschatus *does possess the ability to scavenge this reactive oxygen species (ROS). Among the seed extracts, it was found that AMS-I was efficient in quenching the hydroxyl radical formation and expressed as an IC_50 _value of 16.3 μg GAE/mL, followed by AMS-II (IC_50 _= 18.5 μg GAE/mL). The other two extracts AMS-III and AMS-IV also showed significant hydroxyl radical scavenging effect (Table 2; Figure 4a). On the other hand, the leaf extracts were also found to be potent scavenger of OH^-^. The extract AML-I was the most efficient inhibitor and hence, inhibited the formation of hydroxyl radical to 98.5% followed by AML-II and AML-IV respectively. It is worth to mention that ascorbic acid was shown to be weak inhibitor than the extracts tested (Table 2; Figure 4b).

### Inhibition of Fenton's reagent-induced strand breaks in plasmid DNA

**Figure 5:**
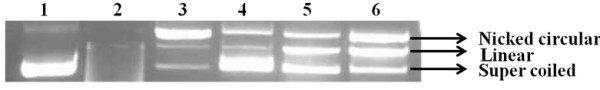
**Effect of seed and leaf extracts of *A. moschatus *on the integrity of pBR322 plasmid DNA in the presence of Fenton's reagents**. (Lane 1: pBR322 DNA + H_2_O; Lane 2: pBR322 DNA + FR; Lane 3: standard antioxidant compound (quercetin) in the presence of FR; Lane 4: pBR322 DNA + FR + AMS-IV; Lane 5: pBR322 DNA + FR + AML-IV; Lane 6: pBR322 DNA + FR + AML-I).

Two seed (AMS-I and AMS-IV) and two leaf (AML-I and AML- IV) extracts have shown significant antioxidant activity in the *in vitro *studies compared to all other extracts. Hence, they were selected for oxidative damage protective activity against a model DNA (pBR322). Hydroxyl radicals generated by Fenton's reaction are known to cause oxidative DNA damage leading to DNA strand breaks and open circular or relaxed DNA forms. This study has revealed that three extracts (AMS-I, AMS-IV and AML-IV) showed effective protection of DNA from damage (nicking) caused by the hydroxyl radicals (Figure 5).

### Inhibition of lipid peroxidation

**Figure 6:**
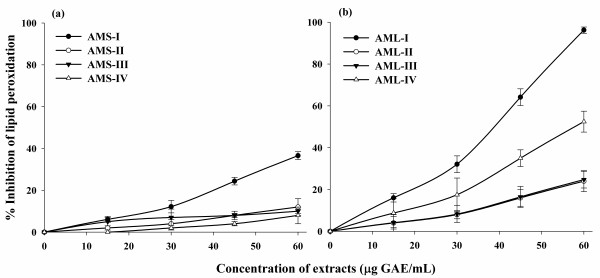
**Inhibition of Lipid peroxidation of seed (a) and leaf (b) extracts of *A. moschatus***.

The percentage inhibition of lipid peroxidation by *A. moschatus *extracts were presented in Figure 6a & Figure 6b and the IC_50 _values were given in Table 2. Compared with the control, AML-I showed significant level of inhibition of lipid peroxidation by 96.2% at 60.5 μg GAE/mL. Other extracts of the leaf (AML-II, AML-III and AML-IV) as well as seed extract (AMS-I) performed poorly in this assay. The seed extracts (AMS-II, AMS-III and AMS-IV) did not exhibit minimal inhibition of lipid peroxidation at the same concentrations.

### Antiproliferative activity

**Figure 7:**
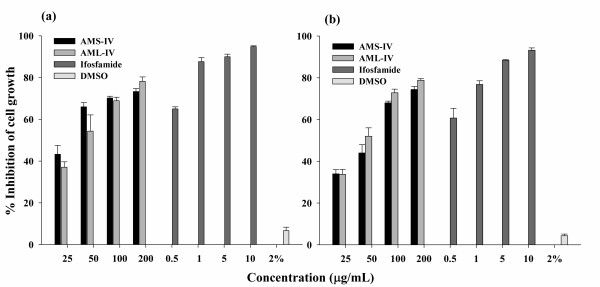
**Antiproliferative activity of *A. moschatus *seed and leaf extracts against COLO-205 (a) and Y79 (b) cell lines**.

The antiproliferative activities of seed (AMS-IV) and leaf (AML-IV) extracts of *A. moschatus *and ifosfamide on the growth of cell lines *in vitro *were presented in Figure 7a and 7b. At the concentration of 200 μg/mL, AMS-IV showed significant antiproliferative activity against the both cell lines COLO-205 and Y79 with the corresponding percentage inhibitory activities of 73.33 ± 1.6 and 74.40 ± 1.6 respectively, under the experimental conditions. Similarly, at the same concentration (200 μg/mL), the leaf extract (AML-IV) showed the 78.25 ± 1.6 and 78.8 ± 0.65 percent inhibitory activity in COLO-205 and Y79 cancer cell lines respectively. Other extracts of seed (AMS-I, II and III) and leaf (AML-I, II and III) did not show any antiproliferative activity on these cell lines.

### Antimicrobial activity

In the present study, the seed and leaf extracts of *A. moschatus *were tested for its antimicrobial activity at various concentrations and evaluated for minimum inhibitory concentration (MIC) values which are presented in Table 3. The extracts showed varying degrees of antimicrobial activity against tested microorganisms. AMS-I and AML-IV extracts exhibited higher degrees of antimicrobial activity than the other extracts. On the contrary, the seed extracts (AMS-II, AMS-III and AMS-IV) and leaf extracts (AML-I, AML-II and AML-III) showed least inhibition of growth of microorganisms. *B. subtilis, S. aureus *and *P. aeruginosa *were most susceptible to AMS-I with MICs value of 101.6 ± 10.2, 109.6 ± 8.3 and 109.6 ± 10.2 μg GAE/mL respectively. Whereas AML-IV has shown strong inhibition with MIC value of 91.7 ± 2.0, 92.0 ± 4.1, 92.5 ± 1.8, 102.6 ± 2.0 μg GAE/mL on *B. subtilis, S. aureus, P. aeruginosa *and *P. vulgaris *respectively. AML- IV also manifested very strong inhibition on growth of *C. albicans *with the MIC value of 45.5 ± 2.2 μg GAE/mL. The inhibitory effects of the extracts were compared with the standard antibiotics such as ampicillin for gram positive bacteria, kanamycin for gram negative bacteria and nystatin for fungal strain. There was no inhibitory effect of ethanol on all tested microorganisms at given concentrations of the solvent.

**Table 3:** Minimum inhibitory concentrations (MIC) of *A. moschatus *extracts against the microorganisms by micro-dilution broth assay

Microorganisms	Seed extracts*				Leaf extracts*				Standard
		
	AMS -I	AMS-II	AMS -III	AMS - IV	AML - I	AML-II	AML-III	AML-IV	
**Gram-positive Bacteria**									
*B. subtilis* ATCC 5740	101.6 ± 10.2	NA	NA	243.5 ± 7.2	NA	NA	NA	91.7 ± 2.0	75.3 ± 2.3^a^
*S. aureus* ATCC 25923	109.6 ± 8.3	323.4 ± 7.1	497.1 ± 6.2	352.5 ± 12.1	NA	195.6 ± 3.3	197.3 ± 4.6	92.0 ± 4.1	65.3 ± 3.7^a^

**Gram-negative Bacteria**									
*E. coli* ATCC 25922	406.41 ± 11.3	948.9 ± 5.4	1543.3 ± 6.4	935.8 ± 2.4	244.4 ± 3.8	195.6 ± 3.9	174.8 ± 9.2	184.2 ± 5.0	102.4 ± 4.7^b^
*P. aeruginosa* ATCC-27853	109.6 ± 10.2	378.7 ± 7.2	485.5 ± 9.3	487.1 ± 3.2	240.2 ± 5.6	197.4 ± 5.0	197.3 ± 4.0	92.5 ± 1.8	67.67 ± 3.8^b^
*P. vulgaris* ATCC 6380	401.7 ± 7.1	948.9 ± 8.4	1375.7 ± 11.0	974.3 ± 4.2	170.7 ± 4.1	195.2 ± 2.0	173.9 ± 2.6	102.6 ± 2.0	58.7 ± 2.9^b^
*S. enterica paratyphi* ATCC 9150	296.7 ± 12.0	714.8 ± 8.8	1063.5 ± 12.4	944.3 ± 3.1	139.9 ± 2.2	194.9 ± 5.5	179.3 ± 4.2	128.6 ± 2.0	35.7 ± 5.6^b^

**Fungi**									
*C. albicans* ATCC 10231	NA	NA	NA	487.1 ± 3.5	NA	NA	NA	45.5 ± 2.2	10.7 ± 0.3^c^

*Values are expressed in μg of GAE/ml; a: ampicillin; b: kanamycin; c: nystatin (μg/ml); Results represented as means ± standard deviation (n = 3); NA: No activity.

## Discussion

Oxidation processes are intrinsic in the energy management of all living organisms and are therefore, kept under strict control by several cellular mechanisms [33]. However, the aberrant production and unbalanced mechanisms of antioxidant protection leads to several human diseases and conditions such as cancer, diabetes, inflammatory disorders, as well as aging processes etc. [34,35]. Natural antioxidants, which are ubiquitous in fruits, vegetables and medicinal plants, have received great attention and have been studied extensively, since they are effective free radical scavengers and are assumed to be less toxic than synthetic antioxidants [36]. The present study is a step towards the exploration of natural antioxidants from seed and leaf extracts of *A. moschatus *employing free radical scavenging assays in addition to anti-proliferative and antimicrobial activities.

Among the various natural antioxidants, phenolics are very important constituents because of their multiple biological effects and direct contribution to antioxidative activity [28]. The results of our study reveal that there is a strong coincidence between antioxidant activity and phenolic content. Several studies on total phenolic content had been published over the years demonstrating its importance in the medicinal field [37-39].

In the present study, antioxidant activity in AMS-I and AML-IV suggests that polyphenols are largely contributing to the total antioxidant activity of these extracts. It is found that the highest antioxidant activity, measured as total antioxidant activity (TAA) values depends on quantities of total polyphenols. Similar results have been published earlier also suggested a causative relationship between total polyphenol content and antioxidant activity [40,41]. Our study indicates that polyphenol present in the extracts of *A. moschatus *might be responsible for the antioxidant properties. Since the antioxidant activity of a substance is usually correlated directly to its reducing capacity, the FRAP assay provides a reliable method to study the antioxidant activity of various compounds [42]. This method has been frequently used for a rapid evaluation of the total antioxidant capacity of different plant extracts containing flavonoids [43]. As shown in Table 1, the ferric reducing power is higher in AMS-I and AML-IV than the rest of the extracts and show a similar trend for total antioxidant activity and this could be attributed to the presence of antioxidant phytomolecules.

The DPPH radical has been used widely as a model system to investigate the scavenging activities of several natural compounds including phenolic compounds, flavonoids or crude mixtures of plants. The effect of antioxidants on DPPH was thought to be due to their hydrogen donating ability [44]. The DPPH radical scavenging abilities of the *A. moschatus *extracts are observed in all the extracts under study in a concentration dependent manner. They are significantly comparable to that of ascorbic acid (100%) showing that the extracts have proton-donating ability and could serve as free radical inhibitors or scavengers, possibly acting as primary antioxidants. It is clear that the antioxidant activity of *A. moschatus *extracts in DPPH assay increased proportionally to the polyphenol content and same trend was observed in earlier reports where increased antioxidant activities showed linear relationship between DPPH values and total polyphenols [45,46].

Hydrogen peroxide is an oxidant that is being continuously generated in living tissues as a result of several metabolic processes. The detoxification of H_2_O_2 _is vital for preventing it from reacting in damaging Fenton-type reactions, which generate extremely reactive oxygen species including hydroxyl free radical [47]. As shown in Figure 2 and Table 2, *A. moschatus *extracts have an effective radical scavenging activity for H_2_O_2 _in a concentration dependent manner and results reveal that these extracts have significant scavenging character in accordance with the standard, ascorbic acid. Similar results have shown that scavenging of H_2_O_2 _by extracts may be attributed to their phenolics, which can donate electrons to H_2_O_2 _and neutralize it to water [48,49].

Several biological reactions generate superoxide radical which is a relatively weak oxidant and exhibits only limited chemical reactivity. It can also generate more dangerous species, including singlet oxygen and hydroxyl radicals, which cause the peroxidation of lipids, thus study of scavenging of this radical is important [50]. In the present study, the seed extracts of *A. moschatus *are found to be an efficient scavenger of superoxide radical generated in PMS/NADH/NBT assay system and percentage of inhibition increases markedly with the increase in concentrations. It suggests that the extracts are potential scavengers of superoxide anion and possibly renders them as promising antioxidants (Table 2; Figure 4). It has also been reported that antioxidant properties of some flavonoids are effective mainly via scavenging of superoxide anion radical [51].

The hydroxyl radical is said to be detrimental and initiates auto-oxidation, polymerization and fragmentation of biological molecules [47,52]. The identification of compounds that have excellent hydroxyl scavenging activity would be significant for some diseases caused by oxidative stress. It has been demonstrated that plants contain many natural antioxidants compounds which have been identified as hydroxyl radical scavengers [53]. Therefore, OH^- ^scavenging effects of *A. moschatus *extracts are assessed in the present study. The result shows that the scavenging activity of both seed and leaf extracts are significantly higher than those of ascorbic acid (Table 2). Hence, *A. moschatus *extracts can be used to minimize the adverse effects from the hydroxyl radicals.

Hydroxyl radicals are also known to be the most reactive species, causes damage to DNA, protein and other life essential biological molecules, leading to mutagenesis, carcinogenesis, and aging [28]. DNA guanosine residues are attacked by hydroxyl radicals generated from Fenton reactants, resulting in strand breakage and transformation from native circular DNA to nicked open circular or relaxed forms. Polyphenols are potential protecting agents against the lethal effects of oxidative stress and offer protection of DNA by chelating redox-active transition metal ions [54]. AMS-I, AMS-IV and AML-IV show effective reduction in the formation of nicked DNA and increased super coiling of DNA (Figure 5). Similar studies have been performed and reported on the protection of DNA by different medicinal plants, thereby confirming antioxidant properties [55,56].

Oxidative damage to cellular components such as cell membrane by free radicals is believed to be associated with pathology of many diseases and conditions including diabetes, cancer, ageing, cardiovascular diseases and inflammatory conditions [57]. One of the degradation products of lipid peroxidation is malondialdehyde (MDA) which causes cell damage and form a pink colour chromogen with thiobarbituric acid. Antioxidants may offer resistance against the oxidative stress by scavenging the free radicals, inhibiting the lipid peroxidation through many other mechanisms and thus prevent diseases [58,59]. Therefore, the inhibition of lipid peroxidation is considered to be important index of antioxidant activity. In our study, the leaf extract (AML-I) shows very strong inhibition of MDA formation (96.2% at 16.5 μg GAE/mL), compared to the other extracts of leaf as well as seed, proving that this extract offers a good degree of protection against the biological end point of oxidative damage.

There has been a 22% increase in cancer incidence and mortality, with over 10 million new cases and over 6 million deaths worldwide in the year 2000 and cases could further increase by 50% to 15 million new cases in the year 2020 [60]. Colon cancer is rapidly rising and is strongly related to age, with 90% of the cases arising in people who are 50 years or above [61]. It is now the third most common malignant disease in both men and women in Asia [62]. Similarly, retinoblastoma is the most common intraocular tumor of childhood and lead to metastatic disease and death in 50% of children worldwide [63]. The use of medicinal plant and fruit extracts for cancer therapy is rapidly evolving as they are affordable, with limited or no side effects. The active components present in such extracts have been shown to efficiently inhibit the process of multi-stage carcinogenesis in a synergistic manner. The identification and characterization of components with potential anti-cancer activity derived from herbal or medicinal plant extracts has been gaining attention. Earlier reports revealed that the antioxidant activity prevents development of cancers [64-67]. So in this context, we have also examined the antiproliferative ability of *A. moschatus *extracts using two human cancer cell lines, COLO-205 and Y79. We found that the proliferation was inhibited in a concentration dependent manner after the exposure to AMS-IV and AML-IV extracts to these cell lines (Figure 7a & Figure 7b). The cytotoxicity was slightly higher in leaf extract than seed extract in both the cell lines tested. Although, the activity is low in comparison to the standard drug, this may be due to the crude nature of the extracts, which can be further enhanced by the purification. It can be inferred that the hydroalcoholic extracts of seed and leaf of *A. moschatus *might be useful as an antiproliferative agent due to the presence of potent bioactive principles [68].

Furthermore, medicinal herbs had been used in ayuverdic traditional medicine for their effectiveness against wide range of diseases due to the advantage of diverse secondary metabolites such as phenolic compounds including flavonoids, alkaloids and tannins [55-57]. Therefore, we also examined the antimicrobial activity of *A. moschatus *extracts against a panel of seven pathogenic microorganisms. Our results indicate that the different extracts of *A. moschatus *exhibit antimicrobial activity and among them, AMS-I and AML-IV are more effective which signifies the antibiotic nature of these extracts (Table 3). Moreover, our observation suggests that organic solvent extract of leaf (AML-IV) is more efficient than other aqueous extracts. Literature also reveals that organic solvent extraction has been proved to be suitable for antimicrobial activities of medicinal plants [69-71]. It is known that the gram negative bacteria are more resistant than the gram-positive ones [72,73] and our results also demonstrate that all the extracts except AML-IV are less effective to these microorganisms even at higher concentrations. The non-activity of the aqueous extracts against most bacterial strains investigated in this study was also in agreement with previous studies which showed that aqueous extracts of plant generally show little or no antibacterial activities [74].

## Conclusions

The present study indicated that *A. moschatus *contains considerable amount of total polyphenols and flavanoids and exhibited good antioxidant activity by effectively scavenging various free radicals. In addition, it has been demonstrated that *A. moschatus *is a potential antiproliferative and antimicrobial agent. The antioxidant and biological activities might be due to the synergistic actions of bioactive compounds present in them. However, it is still unclear which components are playing vital roles for these activities. Therefore, further studies are still needed to elucidate mechanistic way how the plant contributes to these properties. Phytochemical investigation is also proposed to isolate the active fraction and eventually the pure compound(s) from this plant.

## Competing interests

The authors declare that they have no competing interests.

## Authors' contributions

MZG conceived the study, carried out all the experimentation, acquisition and analysis of data and drafting of the manuscript. LMB assisted with the concept and analysis of data. FA was involved in cell culturing, MTT assay. AKK provided technical support and advice in cytotoxic studies. IAQ helped in nystatin study and revision of the manuscript. IAG conceived, designed and supervised the study and revised the manuscript. All authors have read and approved the final manuscript.

## Pre-publication history

The pre-publication history for this paper can be accessed here:

http://www.biomedcentral.com/1472-6882/11/64/prepub
